# Integrative Interpretation of Cardiopulmonary Exercise Tests for Cardiovascular Outcome Prediction: A Machine Learning Approach

**DOI:** 10.3390/diagnostics13122051

**Published:** 2023-06-13

**Authors:** Nicholas Cauwenberghs, Josephine Sente, Hanne Van Criekinge, František Sabovčik, Evangelos Ntalianis, Francois Haddad, Jomme Claes, Guido Claessen, Werner Budts, Kaatje Goetschalckx, Véronique Cornelissen, Tatiana Kuznetsova

**Affiliations:** 1 Hypertension and Cardiovascular Epidemiology, Department of Cardiovascular Sciences, University of Leuven, 3000 Leuven, Belgium; 2 Stanford Cardiovascular Institute and Division of Cardiovascular Medicine, Stanford University School of Medicine, Stanford, CA 94305, USA; 3 Rehabilitation in Internal Disorders, Department of Rehabilitation Sciences, University of Leuven, 3001 Leuven, Belgiumveronique.cornelissen@kuleuven.be (V.C.); 4 Department of Cardiology, Hartcentrum, Virga Jessa Hospital, 3500 Hasselt, Belgium; 5 Faculty of Medicine and Life Sciences, Hasselt University, 3500 Hasselt, Belgium; 6 Cardiology, Department of Cardiovascular Sciences, University of Leuven, 3000 Leuven, Belgium; 7 Cardiovascular Imaging and Dynamics, Department of Cardiovascular Sciences, University of Leuven, 3000 Leuven, Belgium

**Keywords:** cardiorespiratory fitness, cardiopulmonary exercise test, cardiovascular risk stratification, cardiopulmonary phenogrouping, machine learning

## Abstract

Integrative interpretation of cardiopulmonary exercise tests (CPETs) may improve assessment of cardiovascular (CV) risk. Here, we identified patient phenogroups based on CPET summary metrics and evaluated their predictive value for CV events. We included 2280 patients with diverse CV risk who underwent maximal CPET by cycle ergometry. Key CPET indices and information on incident CV events (median follow-up time: 5.3 years) were derived. Next, we applied unsupervised clustering by Gaussian Mixture modeling to subdivide the cohort into four male and four female phenogroups solely based on differences in CPET metrics. Ten of 18 CPET metrics were used for clustering as eight were removed due to high collinearity. In males and females, the phenogroups differed significantly in age, BMI, blood pressure, disease prevalence, medication intake and spirometry. In males, phenogroups 3 and 4 presented a significantly higher risk for incident CV events than phenogroup 1 (multivariable-adjusted hazard ratio: 1.51 and 2.19; p ≤ 0.048). In females, differences in the risk for future CV events between the phenogroups were not significant after adjustment for clinical covariables. Integrative CPET-based phenogrouping, thus, adequately stratified male patients according to CV risk. CPET phenomapping may facilitate comprehensive evaluation of CPET results and steer CV risk stratification and management.

## 1. Introduction

Cardiovascular (CV) diseases substantially burden societal health and healthcare [1]. We are still in search of tools to timely identify individuals at high risk for CV disease in order to timely initiate risk reducing strategies [2]. The assessment of cardiorespiratory fitness (CRF) may improve CV risk assessment [3]. CRF reflects the ability of the muscles to perform dynamic work, relying on the respiratory and CV systems for the transport of oxygen (O_2_) to, and carbon dioxide (CO_2_) from, working muscles. Low CRF is strongly associated with a higher incidence of adverse CV events [3,4]. Nowadays, cardiopulmonary exercise testing (CPET) allows standardized assessment of CRF in clinical settings. To date, however, more awareness needs to be raised about the potential added value of CPET testing for identification of at risk populations [5].

Currently, clinicians interpret a selection of summary CPET metrics to evaluate CRF and its cardiorespiratory determinants [5,6]. Yet, a more integrative interpretation of CPET metrics may provide better characterization of CRF components and, thus, of CV risk. Integrative interpretation of CRF is, however, challenging, due to the complex interrelationships between the different CPET components, such as gas exchange, and hemodynamic and blood pressure (BP) responses. Yet, unsupervised machine learning (ML) approaches may enable integrative CPET profiling by identifying interaction patterns within the complex clouds of interrelated, large-scale CPET data [7]. In addition, such a computational approach could extract and mark the most informative metrics (‘features’) from the pool of variables arising from CPET.

To date, only a few studies have applied unsupervised ML to identify clinically relevant phenogroups from CPET data. In one study, for instance, four patient groups were identified from 738 patients with exercise intolerance based on a network model of invasively measured CPET variables [7]. As such, non-invasive CPET-based phenomapping for CV risk stratification remains unexplored. Therefore, we applied an unsupervised ML approach to identify clinically distinct phenogroups based on CPET summary metrics from a large patient sample with diverse CV risk profiles. Next, we evaluated the clinical value of CPET-based phenogrouping for prediction of incident CV events.

## 2. Materials and Methods

### 2.1. Study Population

This retrospective study is an iCOMPEER sub-study (“Integrative computer modeling for personalized profiling of CRF and prediction of response to ambulatory cardiac rehabilitation”). The study received approval from the Ethics Committee of UZ/KU Leuven (#S64901). We screened data from 3466 adult patients who underwent a maximal CPET at the University Hospital Leuven (Leuven, Belgium) between April 2010 and October 2020. Patients were referred for CPET for diverse reasons: (a) following recent revascularization, including elective percutaneous coronary interventions (PCIs); (b) for CV risk assessment in hypertension, obesity, or diabetes mellitus; (c) as part of screening before initiating an exercise program; or (d) for differential diagnosis of dyspnea, chronic fatigue syndrome or another exercise-limiting condition. Only the first CPET of each patient was considered.

Patients were excluded from analysis if they (1) were below 18 or above 80 years old; (2) had previous myocardial infarction with an impact on left ventricular function (i.e., ejection fraction < 50%), symptomatic heart failure, congenital heart disease, cardiomyopathy, cardiac surgery (e.g., coronary artery bypass grafting) or an artificial pacemaker; (3) had an autoimmune disease, a malignancy or recreational drug abuse; (4) were pregnant; or (5) did not pass the criteria of a maximal CPET (see Section 2.5). The final study sample included 2280 patients.

### 2.2. Retrospective Data Retrieval

Data was pooled from the medical repository system of the University Hospital Leuven. The data comprised demographics, anthropometrics, disease history and medication intake as well as CPET summary metrics. Blood test results for renal function (eGFR) and glucose regulation (blood glucose, HbA1c) from less than 1 year before CPET were retrieved to complement the medical data. This biochemical data was available in 1772 patients (77.7%; median time between blood sample and CPET: 1.1 months).

Definitions of the CV risk factors and comorbidities, such as hypertension, chronic kidney disease and diabetes mellitus, are detailed in the Supplementary Material. CV diseases and surgery were determined from the patient’s medical history files and surgery reports (Table S1).

### 2.3. Outcome Collection (Incident CV Events)

To evaluate the performance of the CPET-based phenogrouping model for CV disease prediction, we collected information on incidence of fatal and non-fatal CV events from the medical repository system. CV events were ascertained until December 2022. Fatal and non-fatal CV events included the following: coronary events (myocardial infarction, acute coronary syndrome, angina pectoris or ischemic heart disease requiring coronary revascularization), symptomatic heart failure, valvular heart disease requiring surgical intervention, heart block, pacemaker implantation, incident atrial fibrillation, stroke, transient ischemic attacks, aortic aneurysm, pulmonary heart disease, pulmonary embolism or infarction, arteriosclerosis, other peripheral vascular disease, arterial embolism or thrombosis, and other diseases of arteries and arterioles. A grace period of 3 months for a first CV event was respected to exclude events resulting from residual therapy (e.g., planned PCI for residual lesions).

### 2.4. Spirometry

Spirometry was available for 1756 of the 2280 patients (77.0%) prior to CPET. Variables recorded were the forced expiratory volume in 1 s (FEV1), the forced vital capacity (FVC), and their ratio (FEV1/FVC). The percentage predicted FEV1 and FVC were calculated as the measured FEV1 and FVC relative to the FEV1 and FVC predicted when considering the patient’s age, sex, and height [8].

### 2.5. Cardiopulmonary Exercise Testing

Patients were instructed not to refrain from taking their medications prior to CPET. Patients had performed a CPET in a hospital setting on a cycle ergometer (Ergometrics 800S, Ergometrics, Bitz, Germany) supervised by a physiotherapist or clinician in accordance with the then prevailing recommendations [9]. Each patient performed an incremental exercise protocol, aiming to reach maximum exertion within 8–12 min (20 W + 20 W/min (79.9%), 20 W + 10 W/min (12.2%), 10 W + 10 W/min (6.7%) or another incremental protocol (1.2%)). During the test, all patients were encouraged to reach maximal exertion and instructed to maintain the cycling cadence between 60 and 70 rotations per minute. Exercise testing was performed under continuous ECG monitoring with breath-by-breath analysis of inspired and expired gas (VO_2_, VCO_2_) and minute ventilation. The BP was measured every other minute using an automated BP monitor. The CPET was stopped upon volitional fatigue, when the patient was unable to keep the pedalling rate above 60 rpm or when any AHA termination criterion was reached [10]. After the test, the patient rated the maximal level of perceived exertion on the 6 to 20 Borg scale. The test supervisor determined the point of the second ventilatory threshold (VT2) using the ventilatory equivalent VE/VCO_2_ method [11]. Patients were included in analyses if VT2 and/or a peak respiratory exchange ratio (RER, the ratio of VCO_2_ and VO_2_) above 1.05 occurred [12].

### 2.6. CPET Summary Metrics

We derived CPET metrics at peak exercise, including the load, VO_2_, RER, HR, O_2_ pulse (as VO_2peak_/HR_peak_), systolic BP and ventilation. VO_2peak_ was the highest of the last three consecutive 30-s averages of VO_2_. Peak O_2_ pulse was calculated as VO_2peak_/HR_peak_. We additionally indexed VO_2peak_ and peak O_2_ pulse for body weight in kilograms. The percentage of age-predicted HR was 100 × [peak HR/maximal predicted HR (defined as 220-age)]. We applied sex-specific equations to calculate the predicted peak VO_2_ from age, weight, and height [in men: −69 + 1.48 × age + 14.02 × height + 7.44 × weight − 0.2256 × age^2^; in women: −588 − 11.33 × age + 9.13 × height + 26.88 × weight − 0.12 × weight^2^] and retrieved percentage predicted VO_2_ as 100 × VO_2peak_/predicted peak VO_2_ [13]. The peak metabolic equivalents of Task (METs_peak_) were calculated as VO_2_/kg_peak_/3.5. The VE/VCO_2_ slope was determined by applying linear regression on the ventilation and VCO_2_ tracings recorded during the exercise period until the respiratory compensation point [14]. In addition, we derived the percentage of VO_2_ and HR at VT1 relative to the peak exercise values.

### 2.7. Statistical Analysis

Study data were managed using the REDCap electronic data management tool hosted at KU Leuven [15]. Means and frequencies were compared between males and females using two-sided unpaired *t*-tests and χ^2^ tests, respectively. A *p* value below 0.05 on a two-sided test was considered statistically significant.

### 2.8. Cluster Analysis for CPET-Based Phenogrouping

**Figure 1:**
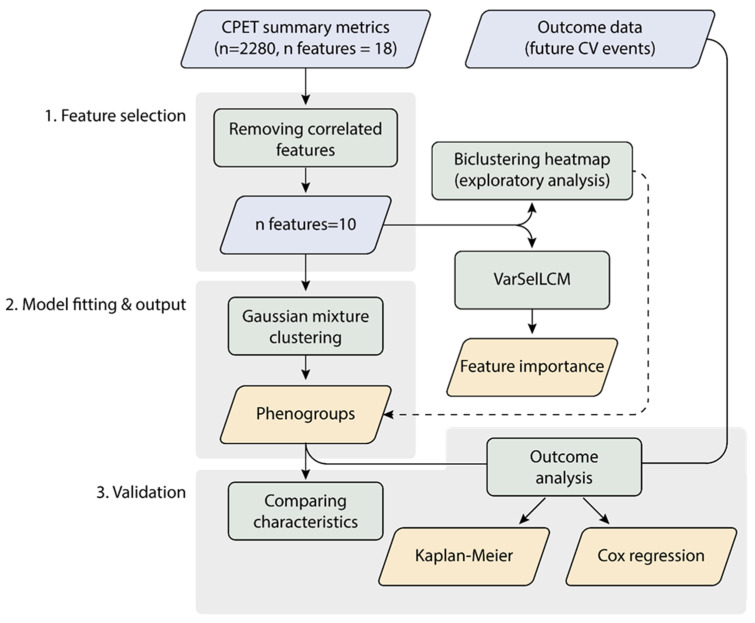
Workflow of the unsupervised machine learning. The blue and orange rhomboids represent the input data and the output of the analysis, respectively. The rectangles describe analytical steps. The dashed line represents data supporting the phenogrouping. CPET, cardiopulmonary exercise testing.

Python 3.8 (https://www.python.org, accessed on 10 May 2023) was used for the unsupervised analysis to identify sex-specific, CPET-based phenogroups and to investigate their associations with the clinical data and CV outcome. Figure 1 summarizes the steps of the unsupervised ML pipeline.

#### 2.8.1. Feature Selection

Before phenogrouping, we applied a feature selection step to avoid any clustering bias originating from strongly correlated features. First, the 18 spirometric and CPET features were standardized to a mean of 0 and standard deviation of 1. A Pearson’s correlation matrix was constructed by using the Pandas library (1.1.2) to identify highly correlated features (i.e., correlations with a Pearson correlation coefficient ≥ 0.8). Of the highly correlated features, only the most connected and most clinically relevant features were kept so as to end up with a set of features for phenogrouping free from strong interrelationships. Eventually, FEV1, FVC, and their ratio were not considered for the phenogrouping as spirometric data was missing in 23% of the patients. In total, 10 CPET features were, thus, selected for phenomapping.

#### 2.8.2. Model Fitting

Two clustering approaches were taken from the scikit-learn library version 0.23, which were applied to the male and female patients separately [16,17]. First, we performed exploratory analysis with biclustering, based on hierarchical clustering with Euclidean distance and Ward linkage, to visually identify patterns that could define subsets of patients based on the previously determined set of 10 features. A biclustering heatmap was created using the seaborn library (v0.10.1), visualizing the grouping of both patients and CPET variables.

Second, we used Gaussian mixture model-based clustering, fit with an expectation maximization algorithm, for the actual CPET-based phenomapping [17,18]. The advantages of Gaussian mixture model-based clustering, compared to other approaches, relate to the following: (i) the possibilities of statistical analysis using the underlying probabilistic framework, (ii) estimating cluster parameters from soft assignments, (iii) accounting for variance and (iv) the formation of clusters of different sizes and shapes [19].

By using Bayesian information criteria (BIC), the ideal number of clusters (K) in men and women was determined for every tested k, as implemented in the scikit-learn library [16,20]. The K with the lowest BIC represented the most appropriate number of clusters. Based on visual inspection of the biclustering heatmap and the BIC values, we instructed the Gaussian mixture algorithm to identify four male and four female phenogroups. Four clusters were considered per sex to enable sufficient granularity between the phenogroups, while maintaining adequate interpretability. Next, the R package VarSelLCM v2.1.3 (https://www.r-project.org, accessed on 10 May 2023) was applied to determine the importance of the features in the phenogroups by sex. The package uses a model-based clustering framework by optimizing a modified integrated complete-data likelihood [21]. Radar charts were used to visualize CPET features across the sex-specific phenogroups.

### 2.9. Model Validation

To define the clinical relevance of the CPET phenogrouping, we first compared clinical characteristics across the phenogroups by means of two-sided unpaired *t*-tests and χ^2^ statistics, with Bonferroni correction of *p* values to account for multiple testing. Next, using the lifelines library v0.27, we plotted the event-free survival of CV events per phenogroup and performed pairwise Log-rank tests comparing the individual Kaplan–Meier curves. We also calculated the multivariable-adjusted Cox proportional hazard ratios expressing the adjusted risk for CV events per phenogroup (with phenogroup 1 as reference). We considered the following confounders in Cox regression: age, body height and weight, heart rate, systolic and diastolic BP, antihypertensive drug intake and a history of diabetes mellitus, chronic kidney disease and cardiovascular intervention. We also tested models that additionally accounted for differences in VO_2_/kg_peak_ or the percentage predicted VO_2peak_.

## 3. Results

### 3.1. Population Characteristics

Table 1 presents the clinical and CPET characteristics by sex. Of the 2280 patients, 1092 (47.9%) were female, 1310 (57.5%) had hypertension and 1057 (46.4%) were taking BP lowering medication. Overall, females were younger than males (47.5 ± 13.7 years in women vs. 55.9 ± 13.0 years in men), and had a more favorable CV risk profile and medical history and less medication intake. Apart from a higher HR at rest and at peak exercise in women, men achieved significantly higher CPET values than women, including a higher load, VO_2_, O_2_ pulse, systolic BP, minute ventilation, VE/VCO_2_ slope and RER at peak exercise (*p* ≤ 0.011) (Table 1).

### 3.2. CPET-Based Phenogroups

#### 3.2.1. Feature Selection and Phenogrouping

**Figure 2:**
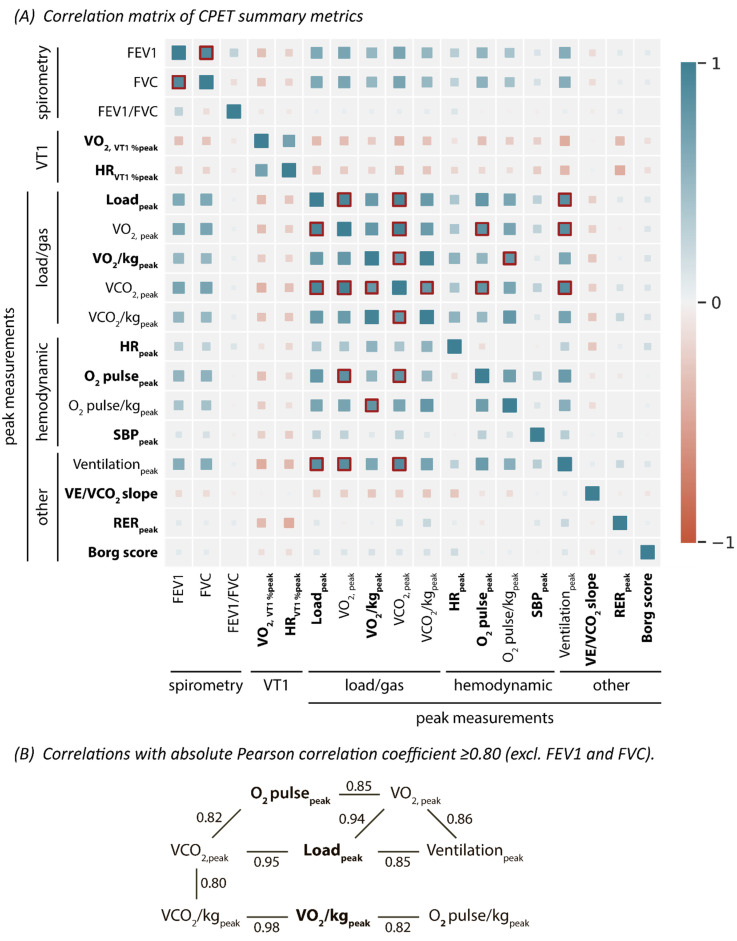
Correlations between the spirometric and the CPET features. Strong correlations, those with an absolute Pearson correlation coefficient > 0.8, were framed in red in the correlation matrix (**A**) and presented below (**B**). The spirometric measurements were eventually excluded from the phenogrouping, due to the extensive amount of spirometric data missing (23.0%). In addition, five CPET metrics were excluded from the phenogrouping due to high collinearity (see B), leaving ten features for the phenomapping (in bold in (**A**)). Abbreviations as in Table 1.

For feature selection prior to the phenogrouping, correlations were determined across 18 spirometric and CPET features by means of a Pearson’s correlation matrix to filter a set of features free from strong interrelationships (Figure 2). Of the eight highly correlated features, we kept load_peak_ and VO_2_/kg_peak_ as features for phenogrouping, given their major clinical importance (Figure 2B). As a result, we excluded VO_2peak_, VCO_2peak_ and peak minute ventilation (because they were strongly correlated with load_peak_) as well as VCO_2_/kg_peak_ and O_2_ pulse/kg_peak_ (because they were strongly correlated with VO_2_/kg_peak_) from the phenogrouping (Figure 2B). In addition, the spirometric variables were also excluded from phenogrouping, as this data was missing in 23% of the patients, leaving 10 CPET variables for phenomapping.

Figure S1 presents the heatmap from biclustering based on agglomerative hierarchical clustering of the 10 CPET features. Based on visual inspection of the biclustering heatmap (Figure S1), and on the BIC values (Figure S2), the optimal number of clusters ranged between 3 and 6 in women and between 4 and 6 in men. Four clusters were eventually considered per sex to enable sufficient granularity between the phenogroups, while maintaining adequate interpretability. As such, the Gaussian mixture modeling algorithm extracted four phenogroups per sex. In both men and women, the load_peak_, VO_2_/kg_peak_, and HR_peak_ had the highest power to discriminate the four clusters (Figure S3). In contrast, SBP_peak_ (and the Borg score in women) were the least important for the phenogrouping.

#### 3.2.2. Comparing Phenogroup Characteristics

**Figure 3:**
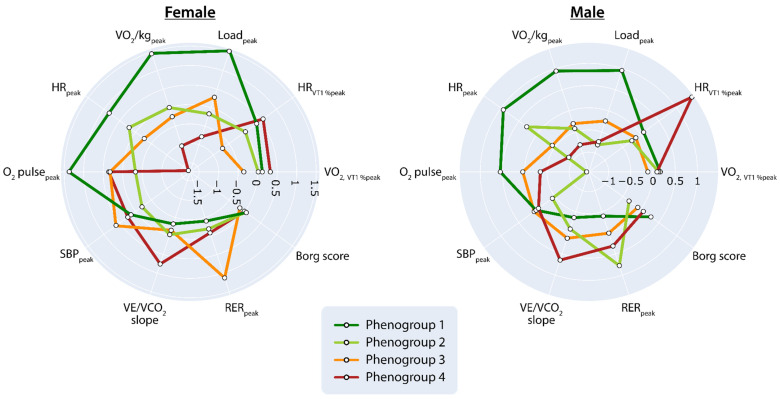
Radar charts of the CPET features used for the phenogrouping illustrate the superposition of these features in each of the four phenogroups identified for each sex. The plot lines compare the clusters’ standardized values expressed as z-scores relative to the global average (=0) across the ten dimensions used as inputs to the clustering process. Abbreviations as in Table 1.

Figure 3 presents the radar charts comparing the 10 CPET features across the sex-specific phenogroups. In general, in both men and women, VO_2_/kg_peak_ and HR_peak_ gradually declined and VE/VCO_2_ slope gradually increased from phenogroups 1 to 4 (Figure 3). Within each sex, phenogroup 4 was, thus, characterized by the lowest VO_2_/kg_peak_, lowest HR_peak_ and highest VE/VCO_2_ slope.

Table S2 presents the clinical characteristics per female phenogroup. From the first (*n* = 102) to the second (*n* = 713) to the third (*n* = 166) to the fourth phenogroup (*n* = 111), we noted the following: (i) a progressive increase in age, BMI and resting systolic BP (ii) a strong increase in disease prevalence (as the history of hypertension, diabetes mellitus, chronic kidney disease and CV disease), (iii) a progressive increase in the intake of antihypertensive, lipid-lowering, anti-thrombotic and anti-diabetic drugs, and (iv) a progressive decline in the FEV1 and FVC (Table S2). In men, we observed the same inter-phenogroup differences, in age, systolic BP, disease history, medication intake and spirometry from the first (*n* = 327) to the second (*n* = 132) to the third (*n* = 661) to the fourth phenogroup (*n* = 68), as in women (Table S3).

#### 3.2.3. The Association between CV Outcome and Phenogroup Assignment

**Figure 4:**
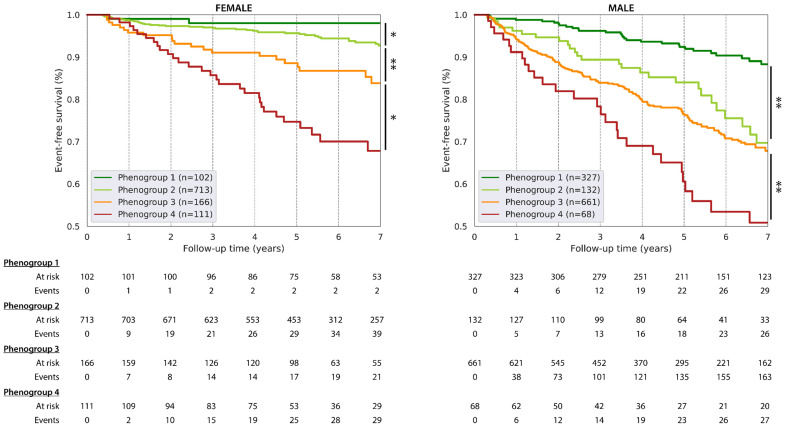
The incidence of cardiovascular events by the integrative CPET profiles. * and ** respectively indicate a *p* value below 0.05 and below 0.001 for a pairwise logrank test.

The median follow-up time was 5.3 years (5th–95th percentile, 1.3 to 10.8 years). During the follow-up time, 278 males and 109 females experienced a CV event (43.9 and 16.2 events per 1000 person-years, respectively). In both sexes, the incidence of CV events increased significantly from phenogroup 1 to 4 (Figure 4).

**Figure 5:**
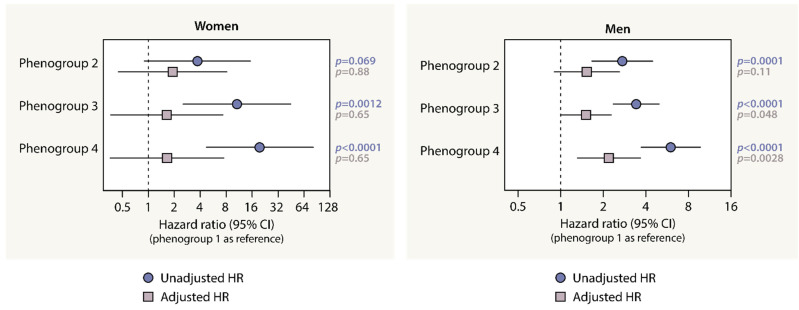
The risk for cardiovascular events by the integrative CPET profiles. The adjusted hazard ratios (95% CI) accounted for differences in age, height, weight, heart rate, systolic and diastolic blood pressure, antihypertensive drug intake and a history of diabetes mellitus, chronic kidney disease, and cardiovascular intervention at baseline. Table 2 details the hazard ratios and *p* values from the unadjusted and adjusted models, including the models accounting for differences in VO_2_/kg_peak_ or % predicted VO_2,peak_.

In males, relative to phenogroup 1, the adjusted risk for CV events was significantly higher for phenogroups 3 (adjusted hazard ratio with 95% CI (HR_adj_): 1.51, 1.00–2.27; *p* = 0.048) and 4 (HR_adj_: 2.19, 1.31 to 3.66; *p* = 0.0028), after adjustment for clinical confounders (Figure 5). After an additional adjustment for VO_2_/kg_peak_ or percentage predicted VO_2peak_, phenogroup 4 was still at higher risk for CV events than phenogroup 1 (Table 2). Reversely, a lower METs_peak_ was significantly associated with a higher CV risk in men, also after adjustment for clinical phenogroup information, at least for METs_peak_ on a continuous scale (Table S4). In females, the risk for future CV events did not differ between the phenogroups after adjustment for the clinical covariables and VO_2_/kg_peak_ or percentage predicted VO_2peak_. (Figure 5, Table 2), but was also not associated with METs_peak_ after accounting for clinical covariables (Table S4).

## 4. Discussion

We leveraged clinical and CPET data from a large and heterogeneous patient sample to construct integrative cardiopulmonary exercise response profiles for CV risk stratification. In brief, unsupervised machine learning identified four CPET-based phenogroups in both men and women, which stratified the patient sample along CV risk in terms of both prevalent and future CV disease. These findings may pave the way to an integrative CPET interpreter for CV risk stratification and CV event prediction.

Previous studies demonstrated the additive prognostic value of individual CPET metrics, beyond traditional risk factors in individuals with symptomatic diseases [22,23,24]. These studies, however, focused on prognostic adverse events in patients with already established diseases, particularly symptomatic heart failure [24]. Moreover, the studies mostly tested the predictive value of only peak O_2_ consumption and the VE/VCO_2_ slope, by means of traditional regression modeling [24]. More research is warranted to assess the added value of integrative CPET evaluation, especially for the identification of at risk (asymptomatic) populations [25].

Within this context, ML approaches (either supervised or unsupervised) may enable the extraction of clinically meaningful profiles from CPET data for early CV risk stratification. Recently, plenty of studies have explored supervised ML methods for disease diagnosis or prognosis using CPET data in at-risk individuals [26,27,28], but only a limited number of studies have applied unsupervised learning approaches. For instance, a previous study on 1619 patients with chronic heart failure applied a cluster analysis on an extensive set of clinical variables, including exercise performance metrics, such as HR_peak_, VO_2,peak_, RER_peak_ and VE/VCO_2_ slope [29]. They identified four distinct heart failure phenotypes heterogeneous in exercise capability and at risk for all-cause mortality and hospitalization. Unfortunately, the authors did not provide insights into the importance of the variables considered in the clustering, making it impossible to evaluate the contribution of the CPET metrics to the phenogroup assignment and performance.

So far, only one study has applied clustering methods to group patients solely based on CPET summary data. In 738 patients with unexplained dyspnea or exertional intolerance who underwent an invasive CPET (iCPET), the algorithm defined four patient groups based on a preselected set of 10 features [7]. Of note, although the authors adjusted minimally for confounders, the assigned phenogroups enabled prediction of three-year all-cause hospitalization independently of separate invasive measures and the clustering was validated in two external patient cohorts [7]. However, the invasiveness of the CPET protocol applied in the study limits the usefulness of its iCPET risk calculator to symptomatic patients with advanced exercise intolerance and hampers its applicability for early CV risk stratification strategies, particularly in settings with milder patient phenotypes.

In our study, the phenogrouping appeared to stratify the patient sample along CV risk in terms of both prevalent and future CV disease. Indeed, in both males and females, we identified a CPET-based phenogroup of patients with the most favorable CV risk profile as being younger, having a lower BMI, a lower prevalence of hypertension, diabetes mellitus and CV disease, a lower medication intake and the greatest response to exercise compared to the other phenogroups. Besides having the lowest risk for prevalent CV disease, patients within this phenogroup also represented the lowest risk of developing CV diseases in the future, which was likely thanks to their having the highest CRFs and more favorable CV risk profiles at baseline. In contrast, the unsupervised clustering identified a distinct CPET-based phenogroup with patients at high risk for future CV events, as it included older patients with an unfavorable CV risk profile (e.g., more CV risk factors, higher disease prevalence and higher medication intake). Of note, in males, the increased risk for future CV events of this ‘poor performing’ phenogroup was found to be independent of traditional CV risk factors. In both males and females, the clusters between the adequate and poor response groups were at intermediate risk with regards to CPET response, severity of CV risk profile and risk for future CV events.

As a proof of concept, our unsupervised clustering analyses illustrate that integration of CPET summary data may generate clinically useful CRF profiles that can help characterize CV health in asymptomatic individuals at risk for future CV disease. In the past, we applied a similar approach to identify echocardiography-based [18,30] and proteome-based [20] phenogroups of CV health, illustrating the potential of integrative biomarker profiling for better CV disease prediction. In addition, the clustering highlighted the CPET metrics most important for adequate discrimination of patient clusters for CV risk stratification. Indeed, the distinction between the phenogroups was predominantly based on differences in metrics of overall CRF (load_peak_ and VO_2_/kg_peak_) and peak hemodynamic response (HR_peak_), and less on differences in other metrics, such as SBPpeak, RERpeak and Borg score. The non-steered preference of the phenogrouping algorithm for CRF measurements, such as VO_2_/kg_peak_, may explain its capability to predict CV events. Importantly, our findings demonstrated an additive value for integrative CPET profiling for CV risk stratification beyond isolated CPET metrics, as the phenogrouping itself was found to be predictive of CV events beyond highly predictive indexes, VO_2_/kg_peak_, and percentage predicted VO_2,peak_.

### 4.1. Clinical Implications and Perspectives

Our approach may enable a more comprehensive, faster, and more observer-friendly way to evaluate CPET results than current approaches or it may be a complementary tool to current approaches. Future large-scale studies should further evaluate the clinical usefulness of integrative CPET profiling for CV risk stratification. In particular, studies should further unravel the extent to which CPET-based phenomapping complements or supplements current approaches for CV risk stratification. Future work should also consider how our approach can be translated to other patient populations. Validated, integrative CPET phenotyping may serve as a complementary tool for healthcare workers to improve the clinical assessment of CRF and steer clinical decision making. Overall, more research is needed to utilize the rich data originating from clinical exercise tests to the fullest. While our analyses were limited to the most common summary CPET metrics, the interpretation of the complex time series recorded during CPET may further improve assessment of CRF components and, in consequence, the risk for CV events.

### 4.2. Limitations and Strengths

We analyzed a rich resource of CPET data from a heterogeneous group of patients who visited a university hospital with a high standard of care. Still, medical reports remain prone to incomplete reporting of diseases and medication. Although the study sample was heterogeneous, some patient groups may have been underrepresented (selection bias), especially individuals too sick to perform a maximal CPET or too healthy to be prescribed one. Furthermore, CPET was supervised by different clinicians and physiotherapists using three devices during routine clinical practice, so caution is necessary regarding interobserver variability in CPET measurements. Yet, CPET was performed by intensively trained staff of a university hospital with a high standard of care and thorough quality controls were performed on all data, including the CPET metrics. Causality cannot be inferred from this cross-sectional setting. Lastly, the phenogrouping model produced during this study has high commercial potential, but a stringent development procedure for the software to be used as a medical device is required before it can be used as a real-life medical application.

## 5. Conclusions

CPET-based clustering by means of unsupervised machine learning algorithms identified integrative CPET profiles, stratifying a heterogeneous patient sample according to CV risk. Such phenogrouping may enable an integrative interpretation of CPET results, facilitate a more comprehensive assessment of CRF and complement risk stratification strategies in asymptomatic individuals. Future studies should further validate the clinical value of cardiopulmonary exercise response profiles for CV risk stratification in the community and in at-risk patient populations.

## Figures and Tables

**Table 1:** Clinical characteristics of 2280 patients.

Characteristics	Men (*n* = 1188)	Women (*n* = 1092)	*p* Value
*Age and anthropometrics*			
Age, years	55.9 ± 13.0	47.5 ± 13.7	<0.0001
Weight, kg	86.0 ± 14.7	71.0 ± 14.5	<0.0001
Height, cm	176.2 ± 7.1	165.2 ± 7.0	<0.0001
BMI, kg/m^2^	27.7 ± 4.4	26.1 ± 5.3	<0.0001
*Medical history*			
Hypertension, *n* (%)	858 (72.2)	452 (41.4)	<0.0001
Diabetes mellitus type I or II, *n* (%)	166 (14.0)	57 (5.2)	<0.0001
Chronic kidney disease, *n* (%)	59 (5.0)	24 (2.2)	0.0006
Obstructive pulmonary disease, *n*(%)	45 (3.8)	44 (4.0)	0.85
CV disease, *n* (%)	732 (61.6)	205 (18.8)	<0.0001
CV intervention, *n* (%)	702 (59.1)	164 (15.0)	<0.0001
*Medication*			
Antihypertensive drugs, *n* (%)	730 (61.4)	327 (29.9)	<0.0001
Lipid-lowering drugs, *n* (%)	779 (65.6)	251 (23.0)	<0.0001
Anti-thrombotic drugs, *n* (%)	755 (63.6)	233 (21.3)	<0.0001
Antidiabetic drugs, *n* (%)	142 (12.0)	56 (5.1)	<0.0001
Spirometry *			
FEV1, L	3.6 ± 0.8	2.8 ± 0.6	<0.0001
FEV1 %predicted	103.8 ± 16.3	103.8 ± 17.6	0.97
FVC, L	4.7 ± 0.9	3.6 ± 0.7	0.0085
FVC %predicted	106.9 ± 15.2	111.7 ± 17.1	<0.0001
FEV1/FVC (%)	77.3 ± 7.6	78.3 ± 8.2	0.0085
*CPET data at rest*			
HR, bpm	71.8 ± 13.6	80.2 ± 14.3	<0.0001
SBP, mmHg	127.1 ± 19.6	118.3 ± 19.0	<0.0001
DBP, mmHg	78.2 ± 11.6	76.9 ± 11.5	0.0061
*CPET data at peak*			
Load, watt	191.9 ± 50.2	131.3 ± 37.2	<0.0001
VO_2_, mL/min	2143 ± 597	1473 ± 380	<0.0001
VO_2 per kg_, mL/kg/min	25.3 ± 7.5	21.3 ± 6.0	<0.0001
VO_2 percentage predicted_, %	89.9 ± 19.5	88.6 ± 18.3	0.083
HR, bpm	147.9 ± 24.7	156.2 ± 23.9	<0.0001
HR _percentage predicted_, %	90.0 ± 12.4	90.5 ± 11.2	0.36
O_2_ pulse, mL/beat	14.5 ± 3.0	9.5 ± 2.0	<0.0001
O_2_ pulse/kg, mL/beat	0.17 ± 0.04	0.14 ± 0.03	<0.0001
SBP, mmHg	181.9 ± 28.6	159.1 ± 28.4	<0.0001
VE, L/min	82.8 ± 23.1	54.0 ± 13.7	<0.0001
VE/VCO_2_ slope	28.6 ± 4.4	28.1 ± 4.4	0.011
RER	1.18 ± 0.08	1.16 ± 0.08	<0.0001
Borg score	15.9 ± 1.6	16.0 ± 1.8	0.24

Data are presented as mean ± SD or number of subjects (%). *p* values are for differences between men and women. * Data available in 877 men and 879 women. BMI, body mass index; CPET, cardiopulmonary exercise testing; CV, cardiovascular; FEV1, forced expiratory volume in 1 s; FVC, forced vital capacity; HR, heart rate; RER, respiratory exchange ratio; SBP, systolic blood pressure; VCO_2_, volume of carbon dioxide exhaled; VE, minute ventilation; VE/VCO_2_ slope, ventilatory efficiency; VO_2_, oxygen uptake.

**Table 2:** The multivariable-adjusted risk for cardiovascular events by the integrative CPET profiles.

					Adjusted Models			
	Unadjusted Model		Clinical Covariables		Clinical Covariables + VO_2_/kg_peak_		Clinical Covariables + % Predicted VO_2, peak_	
	HR (95% CI)	*p* Value	HR (95% CI)	*p* Value	HR (95% CI)	*p* Value	HR (95% CI)	*p* Value
*Females*								
Phenogroup 2	3.72 (0.90–15.3)	0.069	1.92 (0.45–8.17)	0.88	1.79 (0.40–7.94)	0.77	2.55 (0.66–9.78)	0.17
Phenogroup 3	10.7 (2.54–44.9)	0.0012	1.64 (0.46–7.36)	0.65	1.54 (0.33–7.12)	0.55	2.19 (0.55–8.66)	0.26
Phenogroup 4	19.6 (4.71–81.8)	<0.0001	1.65 (0.36–7.50)	0.65	1.51 (0.31–7.33)	0.51	2.51 (0.60–10.6)	0.21
*Males*								
Phenogroup 2	2.72 (1.66–4.46)	0.0001	1.53 (0.90–2.60)	0.11	1.19 (0.69–2.07)	0.52	1.52 (0.85–2.71)	0.15
Phenogroup 3	3.42 (2.36–4.95)	<0.0001	1.51 (1.00–2.27)	0.048	1.30 (0.84–2.01)	0.24	1.54 (1.00–2.37)	0.051
Phenogroup 4	5.99 (3.70–9.70)	<0.0001	2.19 (1.31–3.66)	0.0028	1.76 (1.00–3.07)	0.048	2.09 (1.20–3.67)	0.0098

Hazard ratios (95% CI) represent the risk for cardiovascular events relative to phenogroup 1. Clinical covariables included age, height, weight, heart rate, systolic and diastolic blood pressure, antihypertensive drug intake and history of diabetes mellitus, chronic kidney disease, and cardiovascular intervention at baseline. Models with VO_2_/kg_peak_ did not include weight. Models with % predicted VO_2, peak_ did not account for age (as already considered in the calculation of the % predicted).

## Data Availability

The data presented in this study are available upon reasonable request from the corresponding author. The data are not publicly available due to privacy and ethical issues.

## Supplementary Materials

The following supporting information can be downloaded at: https://www.mdpi.com/article/10.3390/diagnostics13122051/s1, A Supplemental Data File presenting: Supplemental Methods: Table S1: Cardiovascular Diseases and Surgery. Table S2: Characteristics of female patients by phenogroup. Table S3: Characteristics of male patients by phenogroup. Table S4: Multivariable-adjusted risk for cardiovascular events by peak Metabolic Equivalents of Task (METs). Figure S1. Phenogrouping heatmaps for female (left) and male (right) patients from agglomerative hierarchical biclustering analysis on CPET features. Figure S2. Selection of optimal number of clusters according to the Bayesian Information Criterion (BIC). Figure S3. Discriminative power of the CPET features used for phenogrouping by sex.
