# Supplementary material for: Integrative Interpretation of Cardiopulmonary Exercise Tests for Cardiovascular Outcome Prediction: A Machine Learning Approach
Source: Diagnostics (Basel). 2023 Jun 13;13(12):2051. doi: 10.3390/diagnostics13122051 (PMC10297339; doi:10.3390/diagnostics13122051)

Integrative interpretation of cardiopulmonary exercise tests for cardiovascular outcome prediction: a machine learning approach

SUPPLEMENTAL DATA

Nicholas Cauwenberghs, Josephine Sente, Hanne Van Criekinge, František Sabovčik, Evangelos Ntalianis, Francois Haddad, Jomme Claes, Guido Claessen, Werner Budts, Kaatje Goetschalckx, Véronique Cornelissen, Tatiana Kuznetsova

SUPPLEMENTAL METHODS.

Cardiovascular risk factors and (co-)morbidities. Hypertension was defined as clinically diagnosed hypertension, the intake of antihypertensive medication and/or a seated BP ≥140 mmHg systolic or ≥90 mmHg diastolic. Chronic kidney disease was based on clinical diagnosis and/or an eGFR <60mL/min/kg (calculated by the MDRD formula). Diabetes mellitus (type 1 or 2) was based on clinical diagnosis, the use of antidiabetic drugs (unless used for prediabetes), or an HbA1C ≥6.5% (or blood sugar ≥126 mg/dL if HbA1c was unavailable). CV diseases and surgery were determined from the patient's medical history files and surgery reports (*Supplemental Table 1*).

Table S1. Cardiovascular Diseases and Surgery.

Cardiac	***Vascular***
Diseases:<br>• Acute coronary syndrome<br>• Myocardial infarction (ST elevation MI* or non-ST elevation MI)<br>• Angina pectoris with evidence of myocardial ischemia<br>• Congestive heart failure*<br>• Congenital heart disease requiring surgery*<br>• Cardiomyopathy*<br>• Arrhythmia requiring medical intervention<br>• Atrial fibrillation<br>• Pacemaker*<br>• Pericarditis	*Diseases:*<br>• Aortic dissection*<br>• Aortic aneurysm/stenosis<br>• Peripheral artery disease<br>• Arterial embolism/thrombosis<br>• Pulmonary embolism/thrombosis<br>• Pulmonary vascular disease<br>• Venous disease (e.g., deep venous thrombosis)<br>• Blood disorders (e.g., haemochromatosis)<br>• Cerebrovascular: stroke and transient ischemic attack
Surgery:<br>• Percutaneous transluminal coronary angioplasty<br>• Coronary artery bypass grafting*<br>• Heart valvular surgery: valve repair or replacement*<br>• Heart transplantation*	*Surgery:*<br>• Peripheral vascular surgery: endarterectomy, endovascular aortic repair, percutaneous transluminal angioplasty

Diseases and surgical procedures marked with an asterisk (*) were exclusion criteria for this study.

Table S2. Characteristics of female patients by phenogroup.

Characteristics	Phenogroup 1 (n=102)	Phenogroup 2 (n=713)	Phenogroup 3 (n=166)	Phenogroup 4 (n=111)
Age and anthropometrics				
Age, years	36.3±11.3	45.3±12.7*	54.9±10.7*†	61.2±10.3*†‡
Weight, kg	75.3±18.7	67.8±12.5*	77.1±13.7†	78.4±16.4†
Height, cm	170.2±7.0	165.0±6.7*	165.0±7.0*	162.3±6.1*†‡
BMI, kg/m^2	26.0±6.2	24.9±4.6	28.4±5.3*†	29.7±5.8*†
Medical history				
Hypertension, n (%)	25 (24.5)	215 (30.2)	110 (66.3)*†	102 (91.9)*†‡
Diabetes mellitus type I or II, n (%)	1 (1.0)	20 (2.8)	15 (9.0)*†	21 (18.9)*†‡
Chronic kidney disease, n (%)	0 (0.0)	10 (1.4)	5 (3.0)	9 (8.1)*†
Obstructive pulmonary disease, n(%)	2 (2.0)	37 (5.2)	1 (0.6)	4 (3.6)
CV disease, n (%)	2 (2.0)	70 (9.8)*	69 (41.6)*†	64 (57.7)*†‡
CV intervention, n (%)	0 (0)	47 (6.6)*	58 (34.9)*†	59 (53.2)*†‡
Medication				
Antihypertensive drugs, n (%)	8 (7.8)	133 (18.7)*	91 (54.8)*†	95 (85.6)*†‡
Lipid-lowering drugs, n (%)	1 (1.0)	92 (12.9)*	77 (46.4)*†	81 (73.0)*†‡
Anti-thrombotic drugs, n (%)	3 (2.9)	79 (11.1)*	73 (44.0)*†	78 (70.3)*†‡
Antidiabetic drugs, n (%)	4 (3.9)	17 (2.4)	14 (8.4)†	21 (18.9)*†‡
Spirometry				
FEV1, L	3.3±0.6	2.8±0.6*	2.7±0.5*†	2.2±0.6*†‡
FEV1 %predicted	103.8±16.2	103.1±17.4	108.9±17.2†	100.9±21.3‡
FVC, L	4.2±0.8	3.6±0.7*	3.4±0.7*†	2.9±0.6*†‡
FVC %predicted	113.7±18.2	111.3±16.6	114.5±16.5	108.0±19.4
FEV1/FVC (%)	78.7±8.2	78.3±8.5	79.1±6.5	76.2±8.4‡
CPET data at rest				
HR, bpm	84.2±14.3	82.8±13.6	73.5±12.0*†	70.0±13.6*†
SBP, mmHg	116.8±18.4	115.3±17.5	125.3±18.9*†	128.4±22.7*†
DBP, mmHg	76.4±12.4	76.5±11.2	79.3±11.8†	76.3±11.9
CPET data at peak				
**Load, watt**	188.4±30.4	125.2±31.8*	141.9±29.3*†	102.3±27.2*†‡
VO_2, mL/min	2149±260	1416±301*	1499±322*†	1178±306*†‡
**$VO_{2\ per\ kg}$, mL/kg/min**	30.1±7.1	21.3±4.9*	19.8±4.6*†	15.1±2.8*†‡
$VO_{2\ percentage\ predicted}$, %	116.9±15.5	85.7±15.5*	91.8±15.8*†	76.2±14.3*†‡
**HR, bpm**	176.3±13.6	161.1±19.3*	149.7±19.8*†	116.3±17.3*†‡
$HR_{percentage\ predicted}$, %	96.1±7.1	92.3±9.4*	90.6±10.4*	73.2±10.1*†‡
**O_2 pulse, mL/beat**	12.2±1.6	8.8±1.5*	10.1±2.1*†	10.2±2.2*†
O_2 pulse/kg, mL/beat	0.17±0.04	0.13±0.03*	0.13±0.03*	0.13±0.03*
**SBP, mmHg**	163.1±30.3	153.3±25.9*	176.7±27.3*†	166.0±30.1†‡
VE, L/min	71.8±12.4	51.1±12.0*	60.4±11.9*†	47.2±11.2*†‡
**VE/VCO_2 slope**	26.6±3.3	27.9±4.5*	27.4±3.3	31.4±4.6*†‡
**RER**	1.13±0.05	1.14±0.06	1.24±0.10*†	1.15±0.08*‡
**Borg score**	16.1±1.5	16.1±1.9	15.7±1.6	16.0±1.6

Data are presented as mean±SD or number of subjects (%). CPET metrics used for phenogrouping are marked in bold. Significance of pairwise comparisons after Bonferroni correction (with $P_{corrected}$ = $P_{original}$ x 6): *$P_{corrected}$<0.05 versus phenogroup 1, †$P_{corrected}$<0.05 versus phenogroup 2, ‡ $P_{corrected}$<0.05 versus phenogroup 3. Abbreviations as in *Table 1*.

Table S3. Characteristics of male patients by phenogroup.

Characteristics	Phenogroup 1 (n=327)	Phenogroup 2 (n=132)	Phenogroup 3 (n=661)	Phenogroup 4 (n=68)
Age and anthropometrics				
Age, years	46.1±12.6	58.1±13.4*	59.7±10.6*	62.1±9.2*
Weight, kg	87.9±17.4	73.5±11.9*†	87.5±12.4	86.8±13.9†
Height, cm	179.8±7.2	172.6±6.6*	175.2±6.5*†	174.4±6.3*
BMI, kg/m^2	27.1±4.9	24.7±4.1*	28.5±3.9*†	28.5±4.3†
Medical history				
Hypertension, n (%)	156 (47.7)	89 (67.4)*	551 (83.4)*†	62 (91.2)*†‡
Diabetes mellitus type I or II, n (%)	21 (6.4)	20 (15.2)*	110 (16.6)*	15 (22.1)*
Chronic kidney disease, n (%)	3 (0.9)	6 (4.5)	42 (6.4)*	8 (11.8)*
Obstructive pulmonary disease, n(%)	9 (2.8)	4 (3.0)	26 (3.9)	6 (8.8)
CV disease, n (%)	103 (31.5)	76 (57.6)*	495 (74.9)*†	58 (85.3)*†
CV intervention, n (%)	95 (29.1)	73 (55.3)*	479 (72.5)*†	55 (80.9)*†
Medication				
Antihypertensive drugs, n (%)	104 (31.8)	70 (53.0)*	497 (75.2)*†	59 (86.8)*†‡
Lipid-lowering drugs, n (%)	120 (36.7)	86 (65.2)*	514 (77.8)*†	59 (86.8)*†
Anti-thrombotic drugs, n (%)	107 (32.7)	80 (60.6)*	509 (77.0)*†	59 (86.8)*†
Antidiabetic drugs, n (%)	23 (7.0)	14 (10.6)	96 (14.5)*	9 (13.2)
Spirometry				
FEV1, L	4.1±0.7	3.4±0.7*	3.4±0.7*	3.3±0.7*
FEV1 %predicted	105.1±15.3	104.2±17.6	103.1±16.2	102.4±18.8
FVC, L	5.3±0.9	4.4±0.9*	4.4±0.8*	4.4±1.0*
FVC %predicted	110.5±13.7	107.1±17.2	104.9±14.7*	106.5±19.9
FEV1/FVC (%)	77.2±8.1	77.3±7.4	77.5±7.4	75.7±8.2
CPET data at rest				
HR, bpm	77.1±14.0	77.5±14.5	68.0±11.6*†	72.6±5.9‡
SBP, mmHg	122.5±17.9	126.9±20.3	129.1±19.7*	129.7±21.8*
DBP, mmHg	78.8±11.4	77.2±12.2	78.2±11.5	77.4±12.2
CPET data at peak				
**Load, watt**	239.9±49.6	151.5±30.2*	179.9±35.0*†	155.3±39.8*‡
VO_2, mL/min	2758±546	1592±296*	1996±404*†	1690±465*‡
**$VO_{2\ per\ kg}$, mL/kg/min**	32.4±8.3	22.2±5.2*	23.1±4.9*	19.4±4.0*†‡
$VO_{2\ percentage\ predicted}$, %	104.4±19.4	74.1±14.4*	87.4±15.8*	75.6±16.4*‡
**HR, bpm**	170.9±15.4	154.9±18.0*	137.3±20.9*†	126.0±20.5*†‡
$HR_{percentage\ predicted}$, %	98.5±7.7	95.9±9.5*	85.7±11.9*†	79.9±12.4*†‡
**O_2 pulse, mL/beat**	16.1±2.9	10.3±1.3*	14.6±2.5*†	13.4±3.0*†‡
O_2 pulse/kg, mL/beat	0.19±0.04	0.14±0.03*	0.17±0.3*†	0.16±0.03*†‡
**SBP, mmHg**	182.6±30.0	169.6±26.6*	184.1±27.9†	180.6±26.5†
VE, L/min	98.7±24.3	66.3±17.4*	79.4±19.2*†	71.7±19.1*‡
**VE/VCO_2 slope**	27.0±4.0	28.1±4.0*	29.1±4.1*	31.4±7.4*†‡
**RER**	1.15±0.06	1.24±0.10*	1.18±0.07*†	1.20±0.11*
**Borg score**	16.4±1.4	15.4±1.8*	15.8±1.5*	16.0±1.8

Data are presented as mean±SD or number of subjects (%). CPET metrics used for phenogrouping are marked in bold. Significance of pairwise comparisons after Bonferroni correction for multiple testing (with $P_{corrected}$ = $P_{original}$ x 6): *$P_{corrected}$<0.05 versus phenogroup 1, †$P_{corrected}$<0.05 versus phenogroup 2, ‡ $P_{corrected}$<0.05 versus phenogroup 3. Abbreviations as in *Table 1*.

Table S4. Multivariable-adjusted risk for cardiovascular events by peak Metabolic Equivalents of Task (METs).

	Unadjusted model		Adjusted models: Clinical covariables		Adjusted models: Clinical covariables + integrative CPET profiles	
	HR (95% CI)	*P* value	HR (95% CI)	*P* value	HR (95% CI)	*P* value
Females						
$METs_{peak}$, per 2 mL/kg/min decrease	1.75 (1.51-2.02)	<0.0001	1.05 (0.88-1.25)	0.58	1.06 (0.89-1.27)	0.52
$METs_{peak}$ < 5	2.48 (1.19-5.17)	0.015	1.07 (0.50-2.29)	0.86	1.16 (0.54-2.50)	0.70
$METs_{peak}$ 5-7	8.36 (4.16-16.8)	<0.0001	1.45 (0.65-3.23)	0.36	1.42 (0.63-3.18)	0.40
$METs_{peak}$ >7	*reference*		*reference*		*reference*	
Males						
$METs_{peak}$, per 2 mL/kg/min decrease	1.36 (1.27-1.45)	<0.0001	1.17 (1.08-1.27)	0.0002	1.14 (1.04-1.25)	0.0037
$METs_{peak}$ < 5	1.78 (1.26-2.51)	0.0011	1.11 (0.77-1.60)	0.57	0.95 (0.65-1.40)	0.81
$METs_{peak}$ 5-8	3.45 (2.47-4.82)	<0.0001	1.66 (1.14-2.41)	0.0079	1.38 (0.92-2.06)	0.12
$METs_{peak}$ >8	*reference*		*reference*		*reference*	

Hazard ratios (95% CI) represent either the risk for cardiovascular events per 2 mL/kg/min decrease or the risk for cardiovascular events relative to the group with highest $METs_{peak}$. Clinical covariables included age, height, heart rate, systolic and diastolic blood pressure, antihypertensive drug intake and history of diabetes mellitus, chronic kidney disease, and cardiovascular intervention at baseline. $METs_{peak}$ were calculated as $VO_2/kg_{peak}/3.5$.

Figure S1. Phenogrouping heatmaps for female (left) and male (right) patients from agglomerative hierarchical biclustering analysis on CPET features. Columns represent individuals, rows represent CPET features. Colors indicate the Z-score of the particular feature value per patient (red: increased values, blue: decreased values). Feature clusters were labelled A, B, and C. Abbreviations as in *Table 1*.

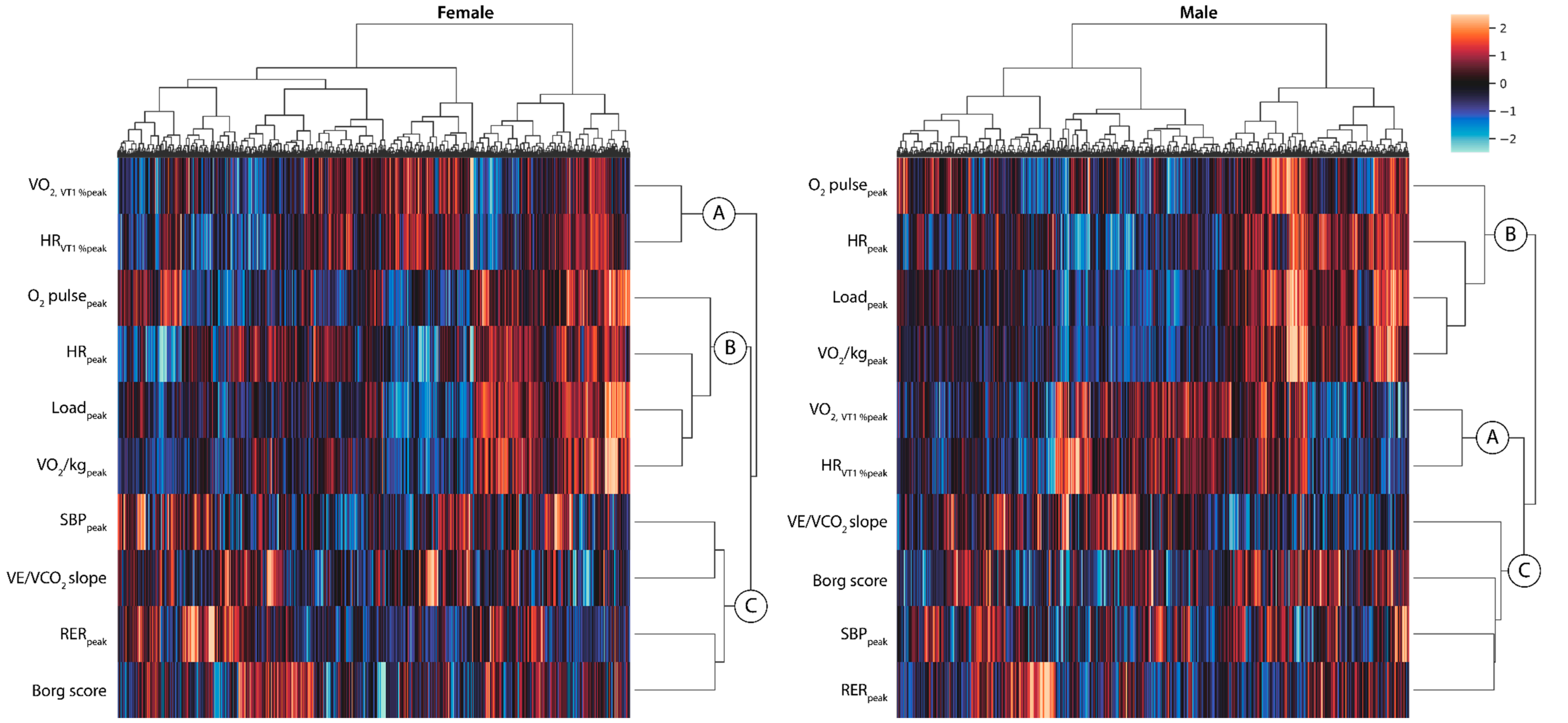

Figure S2. Selection of optimal number of clusters according to the Bayesian Information Criterion (BIC). Values were normalized to range (0, 1). Lower value means better fit. Optimal cluster number ranged between 3 and 6 for women and between 4 and 6 for men. Four clusters were eventually considered per sex to balance granularity and interpretability, while having consistency between men and women.

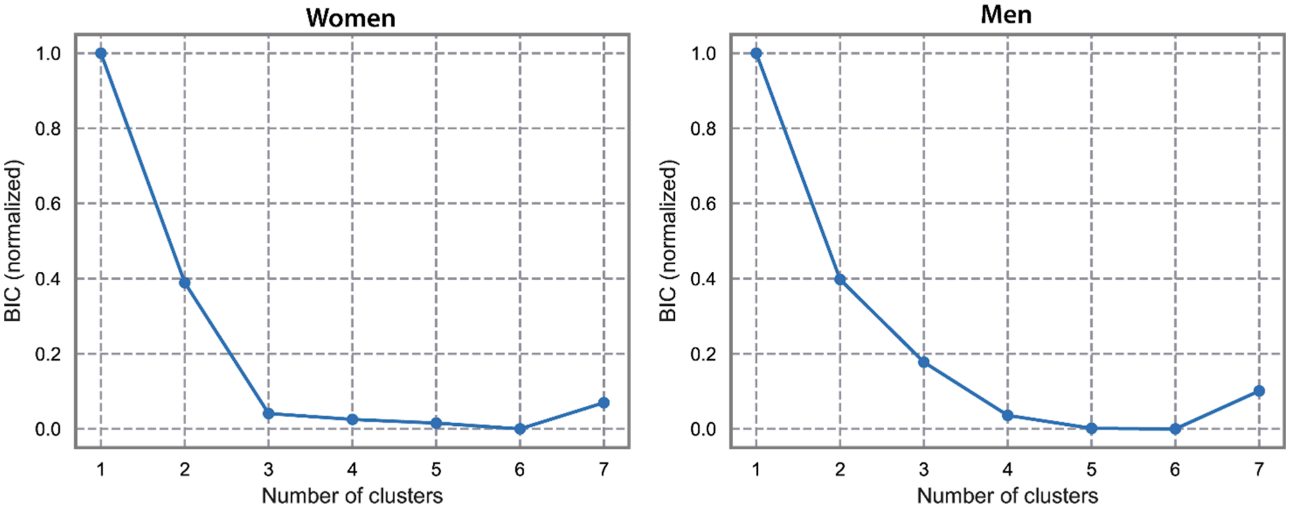

Figure S3. Discriminative power of the CPET features used for phenogrouping by sex. Discriminative power of each feature was defined as the logarithm of the ratio between the probability that the variable is relevant for the clustering, given the best partition, and that the variable is irrelevant for the clustering. The greater the index, the more the variable distinguished the clusters. Abbreviations as in *Table 1*.

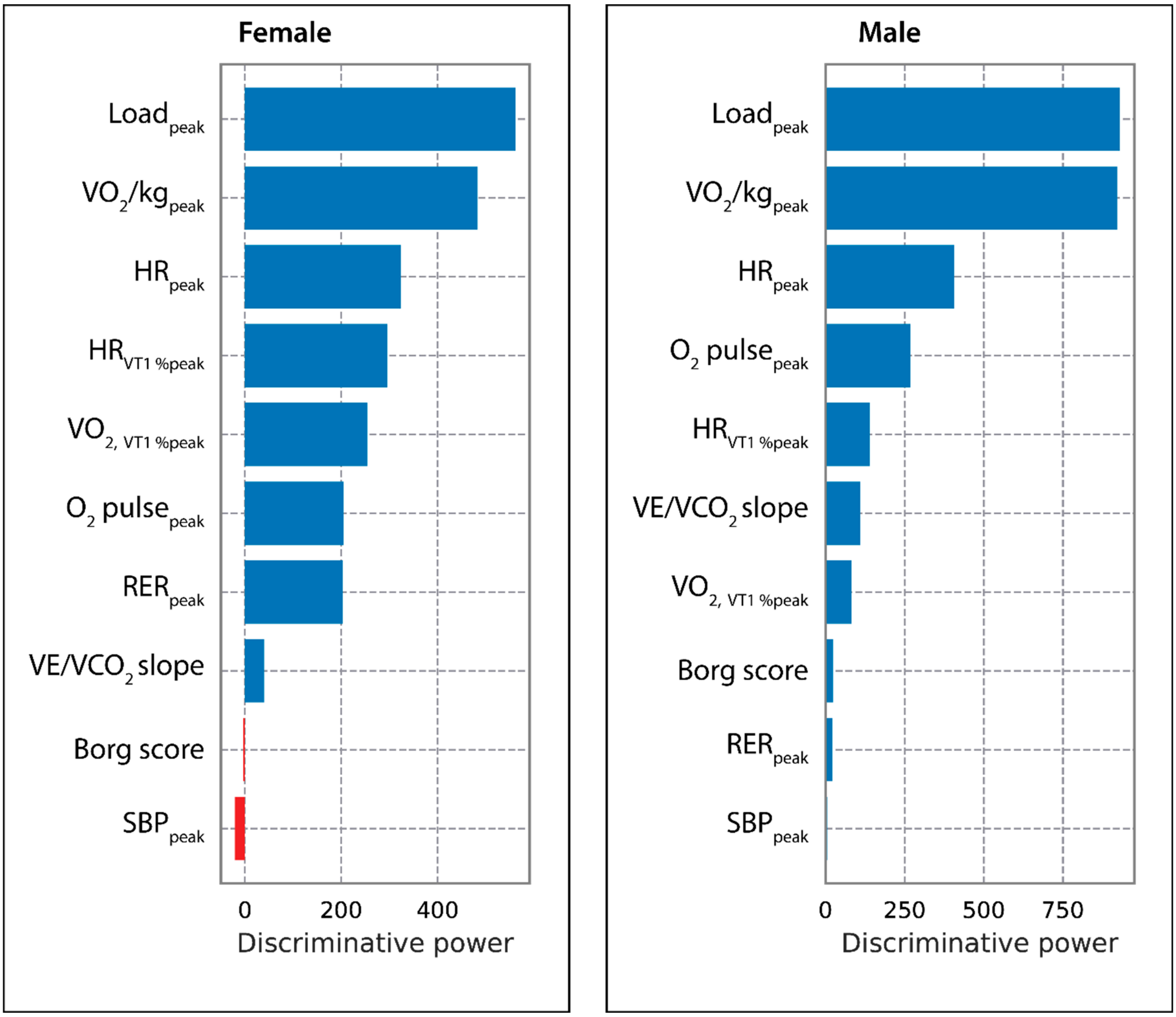

Supplement: Supplementary file 1 [file diagnostics-13-02051-s001.zip › diagnostics-2421503-supplementary.pdf]
